# Post-stroke seizures: classification, risk prediction, and management

**DOI:** 10.3389/fneur.2026.1937496

**Published:** 2026-09-04

**Authors:** Yerko Petar Ivanovic-Barbeito, Kai-Michael Schubert, Anton Schmick

**Affiliations:** 1 Department of Neurology, Tallaght University Hospital, Dublin, Ireland; 2 Department of Neurology, University Hospital Zurich and University of Zurich, Zürich, Switzerland; 3 Department of Clinical and Experimental Epilepsy, UCL Queen Square Institute of Neurology, London, United Kingdom

**Keywords:** acute symptomatic seizure, antiepileptogenesis, classification, patient-centred care, personalised medicine, post-stroke epilepsy, prognostic models

## Abstract

Stroke is the leading cause of adult-onset epilepsy. The International League Against Epilepsy (ILAE) classifies seizures occurring within 7 days of stroke as acute symptomatic seizures (ASyS); later seizures are classified as unprovoked/remote symptomatic. After a single remote symptomatic seizure, a diagnosis of post-stroke epilepsy (PSE) requires an estimated 10-year recurrence risk of at least 60%. We review three frequently conflated questions: how post-stroke seizures should be classified, how the risk of subsequent epilepsy should be estimated, and how patients should be managed. The 2010 ILAE Task Force introduced this definition for epidemiological use, acknowledged that its criteria were somewhat arbitrary, and identified lesion-based refinement in cerebrovascular disease as an unresolved objective; a dedicated ILAE working group is now revisiting the definition. Risk prediction has already progressed beyond a binary approach, largely by incorporating rather than discarding early seizure timing. Temporal classification and multivariable risk estimation are complementary. The principal gap concerns management and diagnostic consequences. Estimated post-stroke seizure risk spans approximately an order of magnitude, whereas the epilepsy label and its driving, occupational, and insurance consequences remain dichotomous. Antiseizure medication prescribing represents a related but distinct problem: treatment should already be individualised, yet available evidence suggests that prescribing is inconsistently calibrated to recurrence risk. Existing prediction models address neither mismatch as they were not designed to do so. We define the evidence required to support a graded framework, the properties such a framework must preserve, and the settings in which its applicability remains unproven.

## 1 Introduction

Stroke is the leading cause of acquired epilepsy in adults (1). Classifying a seizure that occurs soon after stroke as an acute response to the injury or as the first manifestation of epilepsy has implications for the initiation and duration of antiseizure medication, driving eligibility, prognostic counselling, and the patient’s understanding of their condition. The operational distinction is straightforward: a seizure occurring within 7 days of stroke is classified as an acute symptomatic seizure (ASyS), whereas a later unprovoked seizure is remote symptomatic and may satisfy the ILAE definition of epilepsy when the estimated risk of recurrence over the subsequent 10 years is at least 60% (2, 3).

Recent discussions of this rule have often conflated three distinct questions. Although related, these questions have distinct evidence bases. The first concerns classification: which seizures qualify as acute symptomatic. The second concerns prognosis: how the risk of subsequent epilepsy should be estimated for an individual patient. The third concerns management: which clinical or regulatory consequences should follow from classification and prognosis.

First, the available evidence supports retaining the 7-day threshold for classification. Second, prognosis is addressed through validated multivariable models that estimate post-stroke seizure risk on a continuum; each of these models retains early seizure timing as a predictor, making temporal classification and risk modelling complementary. Third, an important management gap persists because the consequences associated with a post-stroke seizure are applied dichotomously, although the underlying risk is continuous. This mismatch, rather than the temporal threshold itself, warrants further investigation. Section 2 describes the review approach; section 3 examines the origin and purpose of the 7-day rule; section 4 summarises the evidence supporting its retention; section 5 discusses its limitations; section 6 reviews current risk-prediction models; section 7 defines the management gap; section 8 outlines the evidence required to address it; and section 9 considers the limitations of the proposed framework.

## 2 Review approach

This is a narrative, hypothesis-generating scoping review. PubMed/MEDLINE and Embase were searched from January 2010, when the ILAE Task Force definition underpinning this discussion was published, through July 2026. Search terms included combinations of “post-stroke epilepsy,” “acute symptomatic seizure”, “SeLECT”, “prognostic model”, “epileptogenesis”, and “antiepileptogenic”; searches were restricted to English-language publications. We also screened reference lists of retrieved articles and relevant ILAE position statements and searched ClinicalTrials.gov and other trial registries for ongoing antiepileptogenesis studies. Articles were selected for conceptual relevance. No protocol was registered, and no formal risk-of-bias assessment was performed; consequently, selection bias cannot be excluded. Statements that no study was identified reflect this search strategy and should not be interpreted as the result of an exhaustive systematic review.

## 3 The 7-day rule: origin and intended purpose

The distinction between a seizure provoked by an acute brain injury and the onset of epilepsy predates the 7-day rule by several decades. Before the 1980s, operational windows ranged from 24 h to several weeks, depending on the cohort and research question. Jennett’s studies of non-missile head injury, published in 1969 and 1973, introduced the first explicit 1-week definition of an early seizure (4, 5). The Rochester Epidemiology Project reports of 1993 and 1995 applied the same interval within a provoked-versus-unprovoked framework encompassing stroke, traumatic brain injury, and central nervous system infection (6, 7). Two later Rochester analyses, published in 1998 and 2009, provided the principal empirical basis for the distinction: among 10-year survivors of a first acute symptomatic seizure, the cumulative risk of a subsequent unprovoked seizure was substantially lower than after a first unprovoked seizure and varied markedly according to status epilepticus and aetiology (8, 9). Other cohorts of the same period used different definitions. The Oxfordshire Community Stroke Project (1997) and the Bladin multicentre study (2000) applied a 2-week window (10, 11), whereas the Rochester cerebral infarction study of So and colleagues (1996) retained 1 week (12). This inconsistency impeded comparisons across cohorts and prompted the ILAE Task Force, in 2010, to recommend 7 days for stroke, traumatic brain injury, and anoxic encephalopathy, and 24 h for metabolic and toxic causes (2). Table 1 lists the pre-2010 definitions in chronological order, together with the two sources cited by the Task Force for cerebrovascular disease.

**Table 1:** Pre-2010 approaches to the acute/unprovoked seizure boundary in cohort and population studies.

Year	First author	Cohort/setting	Cut-off used	Rationale	Notes
1969	Jennett	Non-missile head injury (TBI)	1 week	First definition of early (within-week) post-traumatic seizures	Origin of the seven-day early-seizure boundary (4)
1973	Jennett	Head injury; residual risk by fit-free interval	1 week	Extended the early / late distinction to residual risk	One of only two sources the 2010 ILAE Task Force cited for the stroke window (5)
1993	Hauser	Rochester Epidemiology Project, 1935–1984	1 week (24 h for metabolic / toxic)	Operational provoked / unprovoked framework across stroke, TBI and CNS infection	Foundational ascertainment convention; not validated against alternatives (7)
1995	Annegers	Rochester acute symptomatic seizure incidence	1 week	Continued Rochester convention	Cerebrovascular disease among leading causes; lifetime risk approaching that of epilepsy (6)
1996	So	Population-based cerebral infarction (Rochester)	1 week	Continued Rochester convention	Showed marked recurrence-risk asymmetry after stroke (12)
1997	Burn	Oxfordshire Community Stroke Project	2 weeks	Pragmatic epidemiological window for first-incidence ascertainment	Diverged from Rochester convention (10)
1998	Hesdorffer	Acute symptomatic vs. first unprovoked seizure (Rochester)	1 week	Rochester convention	Status epilepticus modifier: 41% vs. 13% 10-year unprovoked-seizure risk (8)
2000	Bladin	Prospective multicentre stroke study	2 weeks	Followed stroke-epidemiology window	Cortical location, haemorrhage and disability predicted late seizures (11)
2003	Lamy	Cryptogenic ischaemic stroke, young adults	7 days	Aligned with emerging consensus	71% of early seizures occurred within 24 h; replicated recurrence asymmetry (18)
2004	Camilo and Goldstein	Narrative review, seizures and epilepsy after ischaemic stroke	1 week	Summarised prevailing practice	The other of the two sources cited by the Task Force for stroke; not a primary validation (13)
2009	Hesdorffer	Static brain lesion cohort	1 week	Pre-ILAE framing	10-year unprovoked-seizure risk 18.7% vs. 64.8%; 33% for stroke; basis of 2010 consensus (9)
2010	Beghi (ILAE Task Force)	Consensus document	1 week (stroke / TBI / anoxic); 24 h (metabolic / toxic)	Harmonisation across epidemiological studies	Formalised the seven-day rule; described its criteria as “somewhat arbitrary” and called for per-insult, lesion-based refinement (2)

Pre-2010 cohorts used at least three early-seizure windows (24 h, 1 week, and 2 weeks), reflecting differences in aetiology and epidemiological convention rather than competing biological measurements. The 2010 ILAE Task Force adopted 7 days as a harmonising definition for epidemiological studies. For cerebrovascular disease, it cited two sources (5, 13), neither of which compared the 7-day interval with alternative stroke-specific thresholds. TBI, traumatic brain injury; CNS, central nervous system; ILAE, International League against Epilepsy.

Three aspects of the 2010 document warrant emphasis. First, its stated purpose was epidemiological. The Task Force wrote that the definition “was created for use in epidemiologic studies” and that acute symptomatic seizures should be “separately categorized for epidemiologic purposes” (2). The objective was to standardise cohorts, not to determine whether an individual patient should be discharged on antiseizure medication. Second, the Task Force did not claim biological precision. It stated that “the criteria for acute symptomatic seizures are somewhat arbitrary” and described the document as “a first attempt to codify the levels of risk associated with a broad spectrum of insults” (2). Describing the 7-day threshold as arbitrary therefore reiterates a limitation acknowledged by its authors. Third, the Task Force explicitly anticipated further refinement: “further definition of timing of seizures in relation to the acute event is necessary, and further refinement may be possible after taking into account clinical factors associated with neurologic insults (i.e., severity of trauma for head injury; area of the lesion for cerebrovascular disease)” (2). Lesion-based refinement in stroke was therefore part of the original research agenda.

The empirical support for the exact interval is more limited than its widespread adoption might imply. For cerebrovascular disease, the 2010 Task Force cited Jennett and colleagues’ 1973 head-injury data (5) and the 2004 narrative review by Camilo and Goldstein (13); neither source compared the 7-day threshold with alternative intervals in stroke. Subsequent cohort studies have generally adopted the threshold by reference, and, to our knowledge, no study has systematically validated it against competing definitions. The few direct comparisons have produced conflicting proposals: 14 days, on the basis of incidence density in a 2018 meta-analysis (14), and 3 days, on the basis of progression risk in a 2021 cohort study (15). Fifteen years after the Task Force document, the evidence has not materially resolved this question. The ILAE has assigned revision of the acute symptomatic seizure definition to a dedicated working group, separate from the 2025 update of seizure classification (16). A related argument was advanced in 2026 for acute status epilepticus, in which structurally and systemically provoked cases are grouped by temporal proximity despite differing mechanisms and outcomes (17).

## 4 Evidence supporting temporal classification

Any appraisal of the 7-day rule must account for the evidence supporting it. Five considerations favour its retention.

First, the distinction between acute symptomatic and unprovoked seizures provides substantial prognostic information. In the Rochester cohort, the 10-year risk of a subsequent unprovoked seizure was 18.7% after a first acute symptomatic seizure and 64.8% after a first unprovoked seizure; among patients with stroke, the corresponding risk after an acute symptomatic seizure was 33% (9). After adjustment for age, sex, and status epilepticus, a first acute symptomatic seizure was associated with a markedly lower rate of subsequent unprovoked seizures than a first unprovoked seizure (adjusted rate ratio 0.2, 95% CI 0.2–0.4) (9). Despite uncertainty surrounding the exact threshold, the two categories identify groups with substantially different prognoses.

Second, post-stroke seizures are strongly concentrated near stroke onset, making the upper boundary of the acute window less consequential for most patients. In a prospective cohort of young adults, 71% of early seizures occurred within the first 24 h (18); in a 9-centre ischaemic stroke cohort, 55% of first acute symptomatic seizures occurred on day 0 (19). These distributions suggest that classification depends specifically on events occurring on days 6 or 7 in only a small proportion of patients.

Third, validated prediction models incorporate rather than replace the 7-day variable. A seizure occurring within 7 days is a component of SeLECT (Stroke severity, Large-artery atherosclerosis, Early seizure, Cortical involvement, Territory of the middle cerebral artery) and of its extensions SeLECT 2.0 and SeLECT-ASyS (19–21); it is one of the six predictors of IsCHEMiA (Infarct size, Cortical involvement, Haemorrhagic transformation, Early seizure, Middle cerebral artery involvement, Age under 65 years) (22); and it contributes to CAVE (Cortical involvement, Age under 65 years, Volume above 10 mL, Early seizure), LEAN (Lobar haemorrhage, Early seizure, Age under 65 years, Neurosurgery), and RISE (premorbid Rankin score, VASOGRADE delayed ischaemia risk, Surgical treatment, Early-onset seizure) (23–25). The discriminatory performance of these models indicates that this operational definition contains prognostic information. Risk modelling therefore cannot be considered a substitute for temporal classification when the models themselves depend on it. SeLECT-EEG is the exception, because it was developed specifically for stroke survivors without an acute symptomatic seizure and substitutes early electroencephalography (EEG) findings for acute-seizure status (26).

Fourth, the rule is reproducible and broadly applicable. It requires neither EEG nor advanced neuroimaging or a risk calculator. It also facilitates comparisons among cohorts, standardises trial eligibility criteria and administrative coding, and can be communicated to patients and applied by licensing authorities or courts in jurisdictions that have adopted it. These properties are clinically important because most patients with stroke are treated outside specialist centres.

Fifth, the classification supports a treatment principle that remains consistent with available evidence. The Task Force reasoned that acute symptomatic seizures are unlikely to recur unless the provoking condition recurs and that most patients therefore require antiseizure medication only in the short term (2, 27). A subsequent prospective multicentre study reported an approximately 11% 12-month risk of unprovoked seizure after a first acute symptomatic seizure of structural aetiology. Most recurrences occurred while patients were still receiving medication, and longer treatment duration was not associated with a lower recurrence risk (28). However, because the study was non-randomised and excluded status epilepticus, these findings do not establish that prolonged treatment is ineffective; they show only that no benefit of longer treatment was observed.

Collectively, these considerations support the 7-day rule as an epidemiological instrument and a clinical default. They do not support its use as the sole determinant of individual decisions concerning medication, driving, or diagnosis, although the rule has increasingly assumed this role in practice.

## 5 Limitations of a single temporal threshold

Three evidence domains illustrate the limitations of applying a single calendar threshold to all patients.

First, risk is heterogeneous within the 7-day window. In a multicentre cohort of 4,552 patients with ischaemic stroke across nine centres, acute symptomatic seizures occurred in approximately 5%. The 10-year risk of PSE ranged from 13% among patients without an ASyS to 41–94% among those with an ASyS, depending on seizure timing and type; a day-0 seizure was associated with a higher risk of PSE than a seizure later in the same week (adjusted hazard ratio 2.3, 95% CI 1.3–4.0) (19). Status epilepticus and focal-to-bilateral tonic–clonic seizures were associated with the greatest risk. However, the direction of the within-week gradient has not been consistent across studies. In an independent cohort, progression risk was highest on days 4–7 rather than days 0–3 (15); differences in case mix and stroke-subtype composition may have contributed. These findings indicate that the 7-day window contains a distribution of risks rather than a homogeneous prognostic category and that seizure timing within the window may provide additional prognostic information.

Second, the biological processes underlying epileptogenesis do not follow a uniform temporal profile across stroke subtypes. Post-stroke epileptogenesis is a multistage process supported to varying degrees by animal models, human imaging, and circulating biomarkers (29–31). Energy failure and glutamate-mediated excitotoxicity develop within minutes to hours in rodent middle cerebral artery occlusion models. During the first week after stroke, particularly after large cortical infarcts, continuous EEG may demonstrate epileptiform abnormalities, periodic discharges, and patterns on the ictal-interictal continuum (32, 33). Blood–brain barrier disruption represents a second pathway. Albumin extravasation activates TGF-beta signalling in astrocytes, impairs Kir4.1-mediated potassium buffering, and lowers the seizure threshold. In rodent neocortex, astrocytic albumin uptake is mediated by TGF-beta receptors, and receptor blockade reduces epileptogenesis (34). In humans, MRI can demonstrate blood–brain barrier disruption early after ischaemic stroke, at a median of approximately 10 h after onset (35). A third pathway is sustained neuroinflammation, involving microglial release of IL-1beta, IL-6, and TNF-alpha and signalling through HMGB1, TLR4, and the NLRP3 inflammasome. These mechanisms are ictogenic in rodent kainate and pilocarpine models (30) and are increasingly supported by human data on inflammatory markers, gliosis, and superficial siderosis (31).

The duration of these processes differs substantially by stroke subtype. In intracerebral haemorrhage, perihaematomal oedema, blood–brain barrier disruption, thrombin accumulation, erythrocyte lysis, and inflammatory infiltration evolve through multiple phases. Oedema expands most rapidly during the first 48 h and peaks at a mean of approximately 12 ± 3 days on serial imaging (36). These findings provide biological plausibility for a prolonged proconvulsant state in selected patients with intracerebral haemorrhage. However, the cited study assessed oedema rather than seizures or epileptogenesis and therefore provides only indirect support for extending the acute window in haemorrhagic stroke. This distinction is important: the evidence is strongest in haemorrhagic stroke and large cortical infarcts with persistent imaging or EEG abnormalities, whereas no human data establish that a proconvulsant state routinely persists beyond the first week after ischaemic stroke.

**Figure 1:**
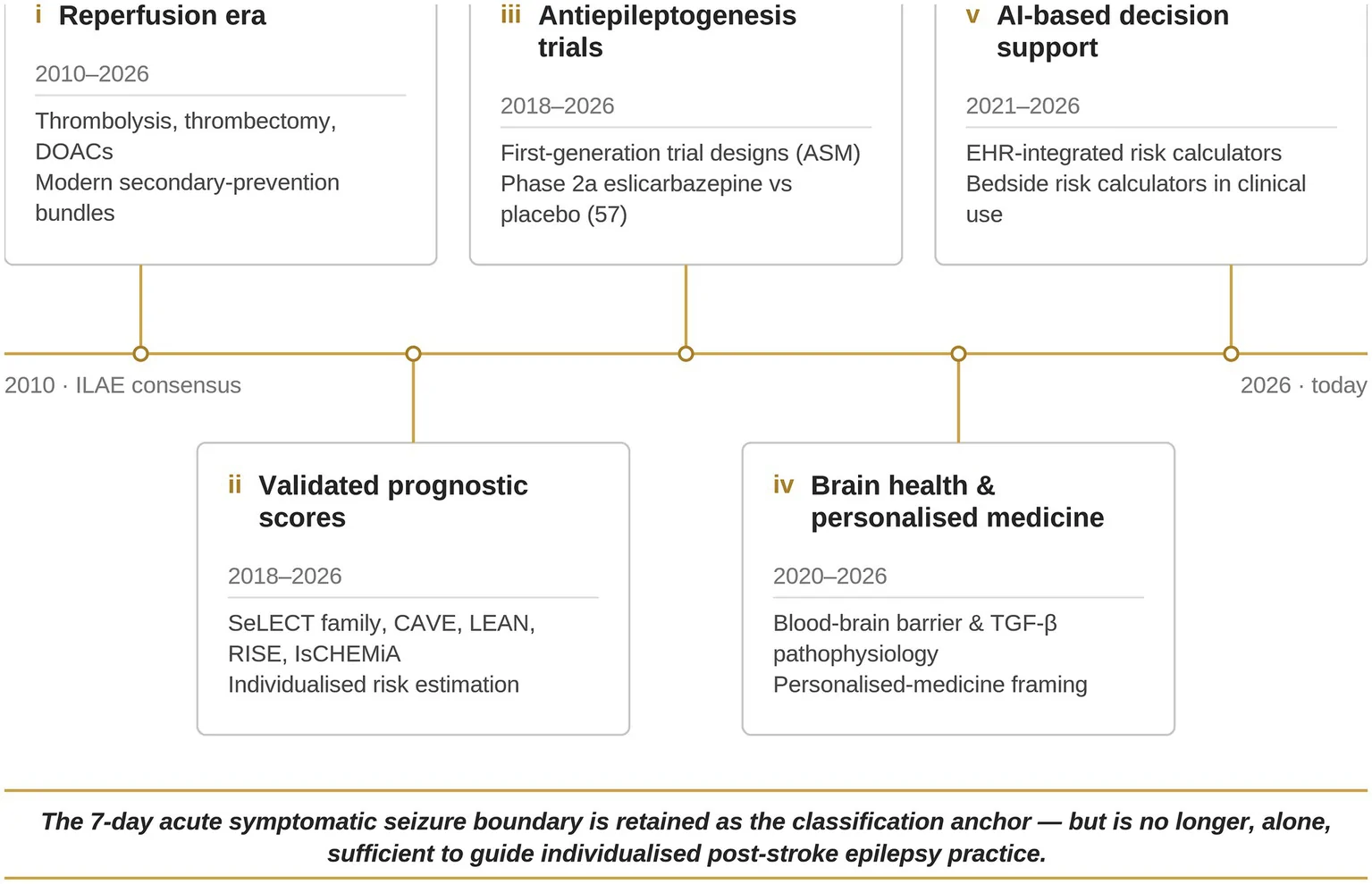
Developments since the 2010 ILAE consensus. The timeline places the 7-day acute symptomatic seizure threshold in the context of subsequent prognostic models, mechanistic studies, and antiepileptogenesis trials. It is intended for orientation rather than as an independent source of evidence; the relevant developments and supporting references are described in sections 3–6 and Tables 2, 3. Era labels indicate the predominant focus of published work during each period and do not imply that any research phase is complete. ILAE, International League against Epilepsy.

Third, the clinical context has changed since 2010. Intravenous thrombolysis, endovascular thrombectomy, contemporary blood-pressure and lipid management, and widespread use of direct oral anticoagulants have altered the populations in which post-stroke seizures occur. Reperfusion, in particular, preserves cortex that would previously have infarcted, modifies haemorrhagic transformation, and changes the temporal evolution of perilesional injury (37). The cohorts underpinning the 7-day rule predate this treatment era. In parallel, antiepileptogenesis has progressed from a mechanistic hypothesis to clinical trials; machine-learning models integrating clinical, neuroimaging, and EEG variables have shown promising discrimination in derivation and external-validation cohorts (38); and web- and smartphone-based prognostic tools are available at the bedside. Figure 1 places these developments in relation to the 2010 consensus. Although none requires abandoning a simple temporal definition, collectively they increase the scope for individualised assessment during the first week after stroke.

## 6 Continuous risk prediction within the temporal framework

Individual risk estimation after stroke has advanced substantially without displacing temporal classification.

Validated prognostic scores combine clinical, neuroimaging, and, when available, electroencephalographic variables to estimate individual PSE risk. Models for ischaemic stroke include the SeLECT family and the imaging-based IsCHEMiA score; CAVE and LEAN were developed for intracerebral haemorrhage, and RISE for aneurysmal subarachnoid haemorrhage (20, 22–25, 39, 40). Tables 2, 3 summarise the derivation and validation data. IsCHEMiA was derived at a single North American centre (n = 1,436) and externally validated in Hong Kong and Japan (n = 2,534). Its c-statistic was 0.87 in derivation and 0.826–0.857 in external validation (pooled 0.85), with estimated 5-year PSE risk ranging from approximately 6 to 78% (22). Early EEG findings provide additional prognostic information: interictal epileptiform discharges, periodic patterns, and lateralised slowing predict subsequent seizures with effect sizes greater than those of most clinical variables (33). SeLECT-EEG is a 0-8-point score developed for stroke survivors without an acute symptomatic seizure. It combines stroke severity measured by the NIHSS, middle cerebral artery territory and cortical involvement, large-artery atherosclerotic aetiology, interictal epileptiform activity, and regional slowing. In the derivation cohort (n = 980; continuous EEG in 336), adding EEG improved discrimination from 0.71 to 0.75. Cumulative PSE risk was approximately threefold higher with than without early epileptiform activity (approximately 42% vs. 13%) (26).

**Table 2:** Validated prognostic models for post-stroke epilepsy after ischaemic stroke.

Feature	SeLECT (20; Lancet Neurol)	SeLECT 2.0 (21; JAMA Neurol)	SeLECT-ASyS (19; Stroke)	SeLECT-EEG (26; Ann Neurol)	IsCHEMiA (22; Neurology)
Acronym meaning	Stroke severity, Large-artery atherosclerosis, Early seizure, Cortical involvement, Territory (MCA)	SeLECT with acute symptomatic status epilepticus added as a covariate	SeLECT updated with the timing (day 0) and type (status epilepticus, FBTCS) of the acute symptomatic seizure	Stroke severity/location + EEG biomarkers, in patients without ASyS	Infarct Size ≥5 cm, Cortical involvement, Haemorrhagic transformation, Early seizure, MCA territory, Age <65 y
Predictor variables	NIHSS (stroke severity); Large-artery atherosclerosis; Early symptomatic seizure; Cortical infarct location; MCA territory	All SeLECT variables; + acute symptomatic status epilepticus (4 additional points)	All SeLECT variables; + ASyS timing (day 0 vs. later) and type (status epilepticus, FBTCS)	NIHSS at admission; MCA territory and cortical involvement; large-vessel atherosclerotic aetiology; interictal epileptiform activity (EEG ≤ 7 days); regional slowing (EEG ≤ 7 days)	Infarct size ≥5 cm; Cortical involvement; Haemorrhagic transformation; Early seizure (≤7 days); MCA territory; Age <65 years
Score range	0–9	0–13	0–7	0–8	0–12 (imaging-anchored variables)
Prediction horizon	1–10 years (5-year commonly reported)	5 years (as reported)	10 years (as reported)	1–10 years	1, 3 and 5 years
Derivation cohort (n)	Swiss stroke registry; *n* = 1,200	9-centre international, 2002–2019; *n* = 4,552 (226 with ASyS, 8 with status epilepticus)	9-centre international, 2002–2018; *n* = 4,552 (233 with ASyS)	Prospective multicentre; *n* = 980 (336 with cEEG)	North American (US); *n* = 1,436
Validation cohort (n)	Austria, Germany, Italy; 3 cohorts, *n* = 1,169	Replication in 3 cohorts; *n* = 39 with acute symptomatic status epilepticus	Switzerland, Argentina, Japan, 2012–2024; *n* = 74 with ASyS	Germany (*n* = 125); Italy (*n* = 84)	Hong Kong + Japan; *n* = 2,534
C-statistic (deriv. / val.)	0.77 (95% CI 0.71–0.82); stable across cohorts	Not reported separately in the source	0.68 derivation (vs 0.58 previous model, *p* = 0.02); 0.70 validation (vs 0.50, *p* = 0.18)	0.71 (SeLECT 2.0 alone); 0.75 (with EEG added)	0.87 (US deriv.); 0.826–0.857 (external); pooled 0.85; vs. SeLECT 0.78 (*p* < 0.0001)
Key risk estimates	Score 0 ≈ 1.3%; Score 9 ≈ 83% (cumulative 5-yr PSE risk)	Score 0 ≈ 2% (95% CI 1–3); score 13 ≈ 100% (95% CI 98–100), 5-year PSE risk	Score 0 ≈ 26% (95% CI 9–39); score 7 ≈ 100% (95% CI 30–100), 10-year PSE risk	Abnormal EEG ≈ 42%; Normal EEG ≈ 13% (cumulative PSE risk)	Score 3 ≈ 6%; Score ≥8 ≈ 78% (5-yr PSE)
Key output(s)	PSE risk over 1–10 years; SeLECT web & app calculator (predictepilepsy.com)	PSE risk over 1–10 years; flags acute symptomatic status epilepticus	PSE risk over 1–10 years; app available	PSE risk over 1–10 years, EEG-enhanced; identifies high-risk EEG subgroup	PSE risk at 1, 3 and 5 years; identifies prophylaxis/trial candidates
Variable type	Clinical + neuroimaging (semi-objective)	Clinical + neuroimaging + seizure type	Clinical + neuroimaging + seizure phenotype and timing	Clinical + neuroimaging + neurophysiology (EEG)	Neuroimaging + early seizure (a clinical variable)
Main limitations	Does not model the competing risk of death; no haemorrhagic-stroke validation	Status-epilepticus stratum rests on 8 derivation cases; wide confidence intervals at the top of the range	Applies only to patients with an ASyS; top stratum rests on few status-epilepticus cases; wide confidence intervals	Requires early EEG (resource-dependent); EEG indication and scoring vary across sites; externally validated only in Germany and Italy to date	Needs detailed neuroimaging; no EEG; no European / African validation

All five tools were derived and validated in cohorts with ischaemic stroke; c-statistics and risk estimates are reproduced from the cited primary studies. SeLECT 2.0 and SeLECT-ASyS are distinct extensions of SeLECT with different additional covariates, score ranges, and reported prediction horizons. Risk estimates should not be compared directly across models because the underlying populations and time horizons differ; absolute estimates should be interpreted within each model. SeLECT-EEG applies only to stroke survivors without an acute symptomatic seizure, whereas SeLECT-ASyS applies only to those with one.

**Table 3:** Validated prognostic models for post-stroke epilepsy after haemorrhagic stroke.

Feature	CAVE (23; Stroke)	LEAN (24; Eur Stroke J)	RISE (25; Neurology)
Stroke subtype	Intracerebral haemorrhage	Intracerebral haemorrhage	Aneurysmal subarachnoid haemorrhage
Acronym meaning	Cortical involvement, Age <65 y, Volume >10 mL, Early seizure	Lobar haemorrhage, Early seizure, Age <65 y, Neurosurgery	premorbid Rankin (R), VASOGRADE scale delayed cerebral ischaemia after subarachnoid haemorrhage (I), Surgical treatment (S), Early seizure (E)
Predictor variables	Cortical involvement; Age <65 y; Volume >10 mL; Early seizure (≤7 days)	Lobar haemorrhage; Early seizure (≤7 days); Age <65 y; any neurosurgical procedure	Premorbid mRS ≥ 2; VASOGRADE-Yellow; Surgical treatment; Early-onset seizures
Score range	0–4	0–4	0–5
Prediction horizon	5 years	3 years (as reported)	5 years
Derivation cohort (n)	Helsinki ICH cohort	South Limburg, Netherlands; *n* = 781 (78 late seizures)	(Vall d’Hebron, 2012–2021); *n* = 419
Validation cohort (n)	Prognosis of InTra-Cerebral Hemorrhage (PITCH) cohort; *n* = 325	External: Lille (*n* = 316), Boston (*n* = 2,052), CROMIS-2 (*n* = 1,042)	External validation *n* = 308
C-statistic (deriv. / val.)	0.81/0.69	0.80 (optimism-corrected, 0.78–0.86); comparable to CAVE on external validation	AUC 0.82 (0.74–0.90)
Key risk estimates	Score 0 ≈ 0.6%; score 4 ≈ 46.2% (derivation; highest stratum small — see limitations). 5-year risk of late seizures	Lowest score ≈ 0.7% to highest ≈ 100% at 3 years; the highest stratum contains few patients and the estimate is correspondingly imprecise	Score 0–1 ≈ 2.9%; score 2–3 ≈ 20.8%; score 4–5 ≈ 75.7% (derivation *n* = 419; 5-year risk)
Variable type	Clinical + neuroimaging	Clinical + neuroimaging	Clinical + imaging grade (VASOGRADE)
Main limitations	Discrimination falls on external validation (c 0.69); needs haematoma-volume measurement	Derivation cohort 2004–2009, predating current ICH management; few late-seizure events (*n* = 78); calibration not established	Single-centre derivation; externally validated

These tools were developed for haemorrhagic stroke, and each includes an early-seizure variable defined by the 7-day rule. None has been validated in ischaemic stroke, and the ischaemic stroke models presented in Table 2 have not been validated in these populations; the two model families should not be used interchangeably. AUC, area under the curve; ICH, intracerebral haemorrhage; aSAH, aneurysmal subarachnoid haemorrhage; mRS, modified Rankin Scale; VASOGRADE, aneurysmal subarachnoid haemorrhage delayed cerebral ischaemia risk grade.

Most models include an early-seizure variable defined by the 7-day rule. SeLECT-EEG is the exception because it replaces acute-seizure status with acute EEG findings in patients without an acute symptomatic seizure. Temporal classification and multivariable risk estimation should therefore not be regarded as competing approaches. Risk models incorporate the temporal variable alongside lesion phenotype, stroke severity, aetiology, and, in some models, EEG. Their contribution is contextualisation rather than replacement of timing: a seizure on day 3 carries different implications after a small subcortical infarct than after a large cortical infarct accompanied by epileptiform discharges, and the models quantify this difference. Clinicians can therefore estimate risk on a continuum without changing the underlying classification.

Two caveats are important. First, these tools predict PSE within the existing framework, but no randomised trial has shown that their clinical use improves PSE outcomes. Furthermore, thresholds for initiating prophylaxis or intensifying counselling remain uncertain (41, 42). Second, the models are heterogeneous and not interchangeable. Models derived for intracerebral haemorrhage should not be applied to ischaemic stroke, and even models for ischaemic stroke require different inputs, ranging from bedside clinical variables to detailed neuroimaging or early EEG (41, 42). Tables 2, 3 therefore present these model families separately.

## 7 Clinical gap: continuous risk and dichotomous consequences

**Figure 2:**
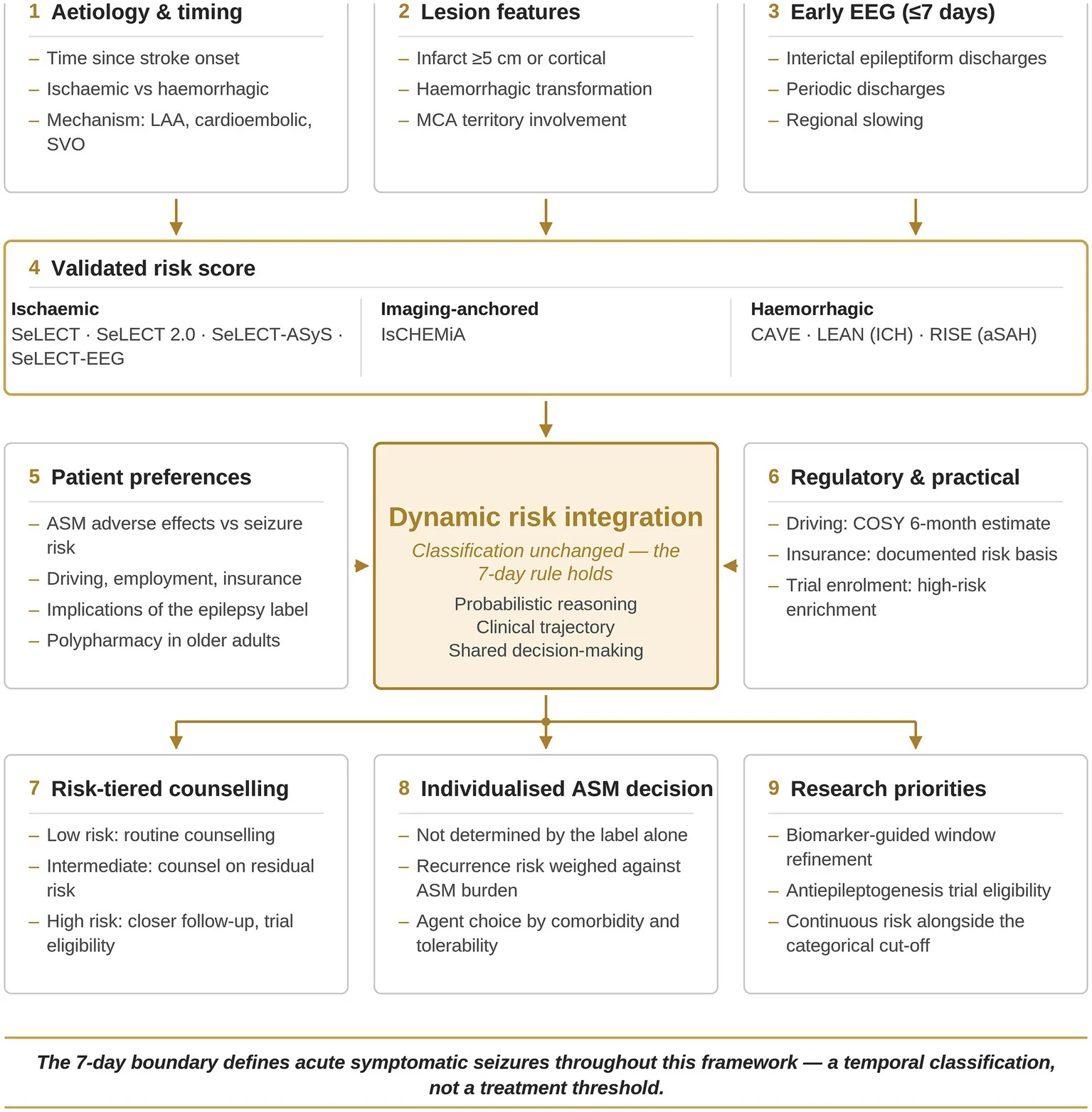
Patient-centred framework for dynamic post-stroke seizure risk assessment and management. Panels (1–3) are the assessment inputs (aetiology and timing, lesion features, and early electroencephalography), which feed the validated risk score in panel (4). Panels (5, 6) are modifiers and constraints (patient preferences; regulatory and practical considerations) that act on the central step, dynamic risk integration. Arrows from that step to panels (7–9) indicate that the integrated estimate informs risk-tiered counselling, the individualised antiseizure medication decision, and research priorities. The 7-day rule remains the temporal classification throughout: Stroke-subtype-specific risk estimates, EEG findings, and patient preferences inform prognosis, counselling, and management but do not reclassify the index seizure. No validated tool currently supports extending or shortening the 7-day interval, and no alternative interval is proposed. The framework is conceptual and identifies relevant inputs without assigning quantitative weights. The 7-day interval remains the clinical default. In high-risk constellations, uncertainty persists as to whether a longer provoked-seizure window is justified. aSAH, aneurysmal subarachnoid haemorrhage; ASM, antiseizure medication; ASyS, acute symptomatic seizure(s); CE, cardioembolic; COSY, Chance Of Seizure in the Year; EEG, electroencephalography; ICH, intracerebral haemorrhage; LAA, large-artery atherosclerosis; MCA, middle cerebral artery; PSE, post-stroke epilepsy; SVO, small-vessel occlusion.

If temporal classification fulfils its intended purpose and risk prediction is already continuous, the remaining gap lies in management: specifically, how risk estimates relate to the consequences of a diagnostic label. We propose neither a new diagnostic interval nor another prediction model. Instead, we outline a research framework for testing whether calibrated risk estimates can improve counselling, follow-up intensity, and treatment review while the 7-day classification remains unchanged. Figure 2 summarises this framework. Aetiology, lesion phenotype, early EEG, validated risk scores, and patient preferences inform a graded assessment of risk and management, whereas the temporal classification remains fixed and regulatory constraints are explicitly acknowledged.

Estimated post-stroke seizure risk spans approximately an order of magnitude: from 5% or less in patients without acute seizures and with small subcortical infarcts to 40–90% in those with day-0 status epilepticus, large cortical or haemorrhagic lesions, and abnormal early EEG (19, 21, 22, 26). By contrast, the consequences associated with a post-stroke seizure are largely categorical. Classification as acute symptomatic or remote symptomatic constitutes the first threshold. Under the ILAE definition, only a remote symptomatic seizure can support a diagnosis of epilepsy after a single seizure, and the estimated 10-year recurrence risk must be at least 60% (3). Once established, the diagnosis may have driving, occupational, insurance, and psychological consequences that do not vary continuously with risk. These consequences are jurisdiction dependent and are not always determined by the epilepsy diagnosis itself; in many jurisdictions, a single seizure triggers driving restrictions regardless of diagnostic classification. A stroke survivor presenting with acute symptomatic status epilepticus may have an approximately 94% 10-year risk of a subsequent unprovoked seizure but cannot be diagnosed with epilepsy on that basis because the index seizure was acute symptomatic (19). Conversely, a patient with one remote symptomatic seizure may receive the diagnosis when the estimated 10-year recurrence risk only marginally exceeds 60%. At equivalent prediction horizons, the first patient may have the greater risk and the second the diagnosis. This distinction does not imply that high predicted risk should retrospectively reclassify the index seizure; it demonstrates that classification and prognosis serve different functions. The 7-day timing criterion and the 60% recurrence-risk criterion therefore create two separate thresholds. Existing prediction models were not designed to reconcile their consequences.

Antiseizure medication prescribing is related but distinct. Temporal classification informs treatment but does not, and should not, determine it. Decisions should integrate EEG findings, lesion characteristics, seizure phenotype, stroke subtype, comorbidities, and estimated recurrence risk. Available evidence nevertheless suggests that prescribing is not consistently calibrated to risk. Antiseizure medication is often initiated after an acute symptomatic seizure and continued for months or years despite limited evidence supporting prolonged treatment. Prospective data indicate that most recurrences occur during treatment and that longer treatment duration is not associated with a lower recurrence risk (28). Undertreatment represents the converse error but appears to affect a smaller group: a day-0 seizure or status epilepticus should prompt heightened surveillance rather than reassurance (19, 21). Both patterns reflect imperfect treatment calibration; neither results directly from the 7-day rule and neither would be corrected simply by changing the threshold.

Current guidelines provide a consistent but incomplete framework for these decisions. On primary prophylaxis the position is uniform. The 2026 American Heart Association/American Stroke Association (AHA/ASA) guideline for acute ischaemic stroke states that prophylactic antiseizure medication is not recommended to prevent seizures or improve functional outcome (Class 3: No Benefit; Level C-LD), while recommending antiseizure medication after an unprovoked post-stroke seizure (Class 1; Level C-LD) on the basis of “specific patient characteristics” (43). The 2022 AHA/ASA intracerebral haemorrhage guideline states that prophylactic antiseizure medication in the absence of evidence of seizures does not improve long-term seizure control or functional outcome (44). The one partial exception is risk-tiered: the 2023 AHA/ASA aneurysmal subarachnoid haemorrhage guideline advises against routine prophylaxis but allows it to be considered in patients with high-risk features, namely a ruptured middle cerebral artery aneurysm, intraparenchymal haemorrhage, high-grade subarachnoid haemorrhage, hydrocephalus, or cortical infarction (45). The European Stroke Organisation (ESO) guideline makes a weak recommendation against primary prophylaxis in both ischaemic and haemorrhagic stroke, citing the low absolute incidence of acute symptomatic seizures (46), and the 2025 Neurocritical Care Society guideline suggests avoiding prophylaxis after non-traumatic intracerebral haemorrhage or, if it is used, preferring levetiracetam over phenytoin and limiting it to 7 days or fewer (47). After an acute symptomatic seizure the guidance is sparser. The ESO guideline suggests not routinely employing secondary prophylaxis, on the grounds that recurrence within the same 7-day window is 10–20% (46), and the subarachnoid haemorrhage guideline recommends treatment for 7 days after a new-onset seizure while advising against continuation beyond that period (45). After a late unprovoked seizure the direction reverses: the ESO guideline notes that observational data place the 10-year recurrence risk after a first post-stroke unprovoked seizure above 70%, comfortably beyond the 60% that the ILAE requires to diagnose epilepsy after a single seizure, and therefore suggests that secondary prophylaxis be considered (46).

Three areas of uncertainty are acknowledged within these documents. First, the quality of the underlying evidence is consistently rated as very low: every ESO recommendation is weak, as are those of the Neurocritical Care Society (46, 47). Second, no guideline specifies how long treatment should continue after an acute symptomatic seizure, and none identifies a preferred agent for established PSE; the AHA/ASA guideline states that data are insufficient to inform the pharmacological management of early seizures at all, and lists the appropriate duration of therapy after a late seizure among its knowledge gaps (43). Third, and most relevant here, none states the level of predicted risk at which management should change. The AHA/ASA guideline identifies early seizure, stroke severity, and cortical involvement as risk factors for an unprovoked post-stroke seizure, three of the five SeLECT variables, but does not link them to a risk estimate or to a treatment threshold (43). The ESO document comes closest, noting as additional information that antiseizure medication is generally not prescribed when the risk of unprovoked seizures does not exceed 50%, and that long-term primary prophylaxis may be an option in patients with intracerebral haemorrhage in whom cortical involvement, age under 65 years, haematoma volume above 10 mL, and an acute symptomatic seizure combine to approach that figure (46). Even there, the estimate is presented as a clinical observation rather than as a recommendation, and no guideline specifies how it should be obtained. The American Academy of Neurology and American Epilepsy Society guideline on a first unprovoked seizure likewise asks clinicians to weigh individualised recurrence risk against the adverse effects of treatment and against patient preference, without defining a threshold or a method of estimation (48). The gap is therefore not that guidelines ignore risk, but that they invoke it without operationalising it.

The choice of antiseizure medication is clinically relevant. Enzyme-inducing agents, including carbamazepine, phenytoin, phenobarbital, and primidone, are associated with greater cardiovascular risk in patients with stroke, partly through interactions with anticoagulants and statins. Non-enzyme-inducing agents are therefore generally preferred in older stroke survivors (49, 50). Selection should be individualised according to renal and hepatic function, cardiac conduction, mood and cognitive effects, hyponatraemia risk, and interactions with anticoagulants and statins. Comparative evidence for individual agents in this population remains limited, and current recommendations are appropriately cautious (49, 50).

Patient preferences are central to management and yet, they remain insufficiently studied. The evidence on treatment preferences and withdrawal cited here was not generated specifically in PSE; its application to this population is therefore extrapolative, and PSE-specific preference research is needed. Patients with stroke are often older and multimorbid and may assign greater importance to avoiding adverse effects than their physicians do (49, 51). Many are also reluctant to discontinue a medication they perceive as effective (52, 53). For a patient whose livelihood depends on a driving licence, minimising recurrence risk may outweigh concerns about adverse effects; another patient may prioritise the opposite balance. Few patients recall receiving numerical risk estimates, although many prefer quantitative information, particularly when presented graphically (52). Dynamic risk tools could help address this communication gap.

The relative weights of its inputs are unknown, and no validated model integrates aetiology, lesion phenotype, EEG, and patient preferences into a single calibrated estimate. Candidate biological markers have reached different stages of development. Genetic predisposition is comparatively well established: a systematic review identified variants in TRPM6, ALDH2, and CD40 associated with higher PSE risk (31), and among 17,549 UK Biobank stroke survivors, the highest decile of an epilepsy polygenic risk score was associated with fivefold higher odds of generalised PSE than the lowest decile (OR 5.05, 95% CI 2.37–12.5) (54). This is a population-level association; no genetic marker has yet been shown to improve discrimination beyond existing clinical models. Markers of inflammation and blood–brain barrier integrity are at an earlier stage. Some are blood based, including IL-1beta, IL-6, HMGB1, and S100B, whereas the serum-to-CSF albumin ratio requires paired cerebrospinal fluid sampling and is not a blood-only assay. None has undergone prospective validation or been implemented as a practical bedside test (31, 38). Figure 2 identifies relevant domains rather than prescribing how they should be combined.

The 60% 10-year recurrence threshold warrants scrutiny. It is a static, population-level criterion for diagnosing epilepsy after a single seizure (3), whereas recurrence risk in acquired epilepsies is greatest during the first 1–2 years and subsequently declines. A fixed threshold incompletely represents this time-varying pattern, and available evidence indicates that remote symptomatic seizures do not consistently exceed 60% across most aetiologies (55). Conceptualising epilepsy as a dynamic condition, with individual risk recalibrated over time, may better reflect the decisions faced by patients (39). Such an approach would complement rather than require replacement of the current definition.

## 8 Evidence required for a graded framework

Three evidence priorities must be addressed before diagnostic or management consequences can be assigned on a graded rather than binary basis.

The first priority is implementation rather than further model derivation. Existing tools were developed predominantly in retrospective cohorts that differed in population and predictor selection, and the models have not undergone external validation to equivalent standards (41, 42). Prospective, standardised validation in the intended settings of use is required, together with real-world evaluation of whether the tools change clinical decisions or outcomes. Bedside implementation is as important as statistical validation. Open platforms such as predictepilepsy.com already provide free access to major post-stroke calculators, including the SeLECT family, IsCHEMiA, CAVE, LEAN, RISE, and COSY (Chance Of Seizure in the Year), for routine counselling (20, 56). The SeLECT Consortium, IPSERC, and large electronic-health-record networks provide infrastructure for international evaluation at scale.

The second priority is trial infrastructure. Future antiepileptogenesis and stroke-recovery trials should incorporate standardised acute assessments as core protocol elements, including harmonised recording of seizure timing, structured early EEG, lesion-phenotype variables, and adjudicated long-term PSE outcomes. A phase 2a, placebo-controlled trial of eslicarbazepine-acetate in patients enriched for PSE risk encountered substantial difficulties with recruitment and event ascertainment and had a null primary endpoint, although it demonstrated the feasibility of this trial concept (57). No antiseizure medication has shown antiepileptogenic efficacy in humans. This experience indicates that risk enrichment is insufficient without reliable biomarkers for case identification. The same infrastructure would strengthen PEPSTEP (perampanel; ACTRN12618001984280), PROPELLER (perampanel; jRCTs051230107), and NCT04858841 (levetiracetam, perampanel, and placebo), particularly if patient-reported and driving-related outcomes are included.

The third priority is prevention. Drug repurposing remains hypothesis generating. Observational and meta-analytic studies have associated statins with lower risks of early post-stroke seizures and PSE (58–61), angiotensin receptor blockers with lower post-stroke epilepsy risk (62–64), and GLP-1 receptor agonists and SGLT-2 inhibitors with lower risks of late-onset seizures or epilepsy (65–69). Supplementary Table S1 summarises the effect estimates, populations, and study designs. Most human evidence is observational and derives from retrospective or propensity-matched cohorts in which residual confounding and confounding by indication remain important. The meta-analysis of randomised trials was not stroke specific, and the preclinical studies in Supplementary Table S1 provide mechanistic rather than clinical evidence. Prospective confirmation is required before these findings can inform practice.

## 9 Limitations of a risk-based framework

Several limitations determine whether the proposed framework can be implemented, irrespective of its conceptual appeal.

The principal limitation is generalisability. Nearly all prognostic tools discussed in this review were derived and validated in high-income health systems, predominantly at tertiary academic stroke centres with access to advanced neuroimaging, continuous EEG, and specialist follow-up. Their discrimination has been established in these environments, which do not represent the settings in which most strokes are managed globally. Performance in lower-resource, non-academic, and low- and middle-income settings remains largely untested. IsCHEMiA, for example, has not been validated in European or African populations, and SeLECT-EEG requires early EEG, which is unavailable in many stroke units (22, 26). A graded framework must therefore specify which patients warrant expanded assessment and which can be adequately managed using the 7-day default; it cannot assume that all inputs are universally available or that model performance is transportable across settings. Simplicity has independent clinical value. A framework that is more accurate under ideal conditions but cannot be implemented or defended at scale would not constitute a practical improvement.

A second limitation concerns the tissue-based analogy underlying this discussion. Post-stroke seizure classification has been proposed to evolve from a time-based to a tissue-based approach, analogous to the transition from a 24-h symptom-based definition of transient ischaemic attack to an imaging-defined criterion (31). This may represent an appropriate long-term objective, but the analogy has an important limitation. Diffusion-weighted imaging directly visualises the infarction invoked by the tissue-based definition of transient ischaemic attack; no equivalent measure exists for epileptogenesis. Epileptiform EEG activity, perilesional imaging abnormalities, and candidate serum biomarkers are correlates of risk rather than direct measurements of the biological process. Tanaka and colleagues similarly note that evidence defining the implementation of a tissue-based approach and its alignment with ILAE criteria remains limited (31). Until suitable biomarkers are validated, tissue-based classification should remain a research objective rather than a clinical template.

A third limitation is the absence of randomised evidence that individualised risk estimation improves outcomes in this population. Although the models show prognostic discrimination, it remains unknown whether acting on their estimates reduces PSE incidence or medication burden or improves driving outcomes or quality of life. Any framework based on these models inherits this uncertainty.

Finally, studies of seizure timing within the acute window are few and heterogeneous in ascertainment. Differences among cohorts may therefore reflect methodology as much as biology. The limitations of our narrative, rather than systematic, review approach are described in section 2.

## 10 Conclusion

The evidence supports distinct conclusions for classification, prognosis, and management. For classification, the 7-day rule should be retained. It was developed as an epidemiological convention and performs that function effectively. Its authors acknowledged its arbitrary elements and anticipated lesion-based refinement, a question now being addressed by an ILAE working group (2, 16). For prognosis, continuous individualised estimation is already possible and has been achieved primarily by incorporating rather than replacing early seizure timing. SeLECT-EEG is the exception because it was developed for patients without an acute symptomatic seizure. Temporal classification and multivariable prediction should therefore be considered complementary (19–22, 26).

For management, a mismatch remains. Risk is estimated on a continuum, whereas the consequences attached to the diagnostic label are applied dichotomously. These include the diagnosis of epilepsy, which is jointly constrained by the 7-day classification and the 60% recurrence-risk threshold, and its potential driving, occupational, and insurance consequences. Antiseizure medication prescribing is a related but separate calibration problem. Current guidelines are consistent in advising against prophylaxis and in favouring treatment after a late unprovoked seizure, and several of them invoke individual recurrence risk, but none specifies the level of risk at which management should change or how that risk should be estimated (43–48). Existing prediction models were not designed to fill that space, and changing the temporal threshold alone would not do so.

The principal remaining evidence gap is therefore one of implementation, and closing it is the research priority we would place first: prospective studies that apply a validated risk estimate in the settings where stroke is actually managed, and that measure whether acting on it changes counselling, follow-up intensity, treatment duration, or driving outcomes, while preserving the simplicity, portability, and defensibility of the current rule. Until such evidence exists, the 7-day threshold should remain the classification standard, used for what it demonstrably does well: it separates two groups with substantially different prognoses (9), it can be applied without EEG, advanced imaging, or a calculator, and it supplies the early-seizure variable on which nearly every validated prognostic model depends (19–25). What it does not do is quantify risk for an individual patient. That is the function of the models, and the two should not be conflated. In the interim, the defensible clinical position is transparency rather than prescription. Features that existing evidence and guidance already treat as high risk - large or lobar haemorrhagic lesions, day-0 seizures or status epilepticus, and persistent early epileptiform EEG abnormalities - may reasonably prompt more detailed counselling, closer follow-up, and, where available, discussion of trial participation, rather than reclassification of the index seizure (19, 21, 23–26, 45, 46). No validated model combines these features into a single calibrated estimate, however, so the resulting judgement remains qualitative; clinicians should say so, and should make explicit that diagnostic classification and predicted risk are related but distinct.
