# Supplementary material for: Post-stroke seizures: classification, risk prediction, and management
Source: Front Neurol. 2026 Sep 4;17:1937496. doi: 10.3389/fneur.2026.1937496 (PMC13587696; doi:10.3389/fneur.2026.1937496)
Supplement: Supplementary file 1 [file Supplementary_file_1.docx]

**Table S1.** Pre-2010 approaches to the acute / unprovoked seizure boundary in cohort and population studies.

| **Year** | **First author** | **Cohort / setting** | **Cut-off used** | **Rationale** | **Notes** |
| --- | --- | --- | --- | --- | --- |
| 1969 | Jennett | Non-missile head injury (TBI) | 1 week | First definition of early (within-week) post-traumatic seizures | Origin of the seven-day early-seizure boundary (4) |
| 1973 | Jennett | Head injury; residual risk by fit-free interval | 1 week | Extended the early / late distinction to residual risk | One of only two sources the 2010 ILAE Task Force cited for the stroke window (5) |
| 1993 | Hauser | Rochester Epidemiology Project, 1935-1984 | 1 week (24 h for metabolic / toxic) | Operational provoked / unprovoked framework across stroke, TBI and CNS infection | Foundational ascertainment convention; not validated against alternatives (7) |
| 1995 | Annegers | Rochester acute symptomatic seizure incidence | 1 week | Continued Rochester convention | Cerebrovascular disease among leading causes; lifetime risk approaching that of epilepsy (6) |
| 1996 | So | Population-based cerebral infarction (Rochester) | 1 week | Continued Rochester convention | Showed marked recurrence-risk asymmetry after stroke (12) |
| 1997 | Burn | Oxfordshire Community Stroke Project | 2 weeks | Pragmatic epidemiological window for first-incidence ascertainment | Diverged from Rochester convention (10) |
| 1998 | Hesdorffer | Acute symptomatic vs first unprovoked seizure (Rochester) | 1 week | Rochester convention | Status epilepticus modifier: 41% vs 13% 10-year unprovoked-seizure risk (8) |
| 2000 | Bladin | Prospective multicentre stroke study | 2 weeks | Followed stroke-epidemiology window | Cortical location, haemorrhage and disability predicted late seizures (11) |
| 2003 | Lamy | Cryptogenic ischaemic stroke, young adults | 7 days | Aligned with emerging consensus | 71% of early seizures occurred within 24 h; replicated recurrence asymmetry (18) |
| 2004 | Camilo and Goldstein | Narrative review, seizures and epilepsy after ischaemic stroke | 1 week | Summarised prevailing practice | The other of the two sources cited by the Task Force for stroke; not a primary validation (13) |
| 2009 | Hesdorffer | Static brain lesion cohort | 1 week | Pre-ILAE framing | 10-year unprovoked-seizure risk 18.7% vs 64.8%; 33% for stroke; basis of 2010 consensus (9) |
| 2010 | Beghi (ILAE Task Force) | Consensus document | 1 week (stroke / TBI / anoxic); 24 h (metabolic / toxic) | Harmonisation across epidemiological studies | Formalised the seven-day rule; described its criteria as “somewhat arbitrary” and called for per-insult, lesion-based refinement (2) |

*Pre-2010 cohorts used at least three early-seizure windows (24 hours, 1 week, and 2 weeks), reflecting differences in aetiology and epidemiological convention rather than competing biological measurements. The 2010 ILAE Task Force adopted 7 days as a harmonising definition for epidemiological studies. For cerebrovascular disease, it cited two sources (5, 13), neither of which compared the 7-day interval with alternative stroke-specific thresholds. TBI, traumatic brain injury; CNS, central nervous system; ILAE, International League Against Epilepsy.*
